# RNA-Seq Analysis Provides Insights for Understanding Photoautotrophic Polyhydroxyalkanoate Production in Recombinant *Synechocystis* Sp.

**DOI:** 10.1371/journal.pone.0086368

**Published:** 2014-01-22

**Authors:** Nyok-Sean Lau, Choon Pin Foong, Yukio Kurihara, Kumar Sudesh, Minami Matsui

**Affiliations:** 1 Ecobiomaterial Research Laboratory, School of Biological Sciences, Universiti Sains Malaysia, Penang, Malaysia; 2 Synthetic Genomics Research Team, Biomass Engineering Program Cooperation Division, RIKEN Center for Sustainable Resource Science, Yokohama, Kanagawa, Japan; University of New South Wales, Australia

## Abstract

The photosynthetic cyanobacterium, *Synechocystis* sp. strain 6803, is a potential platform for the production of various chemicals and biofuels. In this study, direct photosynthetic production of a biopolymer, polyhydroxyalkanoate (PHA), in genetically engineered *Synechocystis* sp. achieved as high as 14 wt%. This is the highest production reported in *Synechocystis* sp. under photoautotrophic cultivation conditions without the addition of a carbon source. The addition of acetate increased PHA accumulation to 41 wt%, and this value is comparable to the highest production obtained with cyanobacteria. Transcriptome analysis by RNA-seq coupled with real-time PCR was performed to understand the global changes in transcript levels of cells subjected to conditions suitable for photoautotrophic PHA biosynthesis. There was lower expression of most PHA synthesis-related genes in recombinant *Synechocystis* sp. with higher PHA accumulation suggesting that the concentration of these enzymes is not the limiting factor to achieving high PHA accumulation. In order to cope with the higher PHA production, cells may utilize enhanced photosynthesis to drive the product formation. Results from this study suggest that the total flux of carbon is the possible driving force for the biosynthesis of PHA and the polymerizing enzyme, PHA synthase, is not the only critical factor affecting PHA-synthesis. Knowledge of the regulation or control points of the biopolymer production pathways will facilitate the further use of cyanobacteria for biotechnological applications.

## Introduction

Cyanobacteria are believed to be one of the oldest groups of photosynthetic organisms on Earth and played a significant role in the development of the oxygenic atmosphere we breathe today [1]. In modern day, cyanobacteria continue to play a pivotal role in global carbon recycling, the nitrogen cycle and most importantly, the maintenance of the composition of the atmosphere [2], [3]. Cyanobacteria are considered to be ideal producers of various fine chemicals and biofuels because they fix carbon dioxide into biomass using solar energy. Fluctuations of nutrient concentrations constantly occur in natural environments and microorganisms respond to nutrient starvation by accumulating various carbon and energy storage compounds [4]. The study of these storage polymers, particularly polyhydroxyalkanoate (PHA), has gained considerable interest in recent years in an attempt to address the waste disposal problems caused by petrochemical plastics [5].

At present, the major biological processes utilized for industrial production of PHA are fermentations of heterotrophic bacteria. Nevertheless, the economic viability of PHA as a commodity polymer is limited by high production costs due to costly carbon substrates and requirements during the fermentation processes. Substantial effort has been devoted to investigating PHA production processes that are more cost-effective [6]. An interesting and promising approach is the use of photosynthetic cyanobacteria as the host for PHA production. The cyanobacteria, as ‘microbial factories’, can fix carbon dioxide from the atmosphere into high molecular weight PHA directly via photosynthesis. Besides being photoautotrophic, cyanobacteria require minimal nutrients for growth, eliminating the cost of carbon sources and complex growth media [7]. Thus, the application of cyanobacteria offers the potential of a cost-competitive and sustainable approach for the production of this environmentally friendly polymer.

The presence of PHA in cyanobacteria was first described by Carr whom analyzed PHA in *Chloroglea fritschii* based on acid hydrolysis of poly(3-hydroxybutyrate), P(3HB), to crotonic acid followed by UV spectroscopic measurement of the hydrolysis product [8]. Since then, much research has demonstrated the presence of PHA in several other cyanobacteria including *Aphanothece* sp. [9], *Oscillatoria limosa* [10], some species of the genus *Spirulina* [11], [12] and the thermophilic strain *Synechococcus* sp. MA19 [13]. So far, cyanobacteria are characterized by their ability to produce PHA containing only 3-hydroxybutyrate (3HB) and/or 3-hydroxyvalerate (3HV) monomers [9], [10], [14]. Although there are many reports on the occurrence of PHA in cyanobacteria, most of these studies explored the physiology and fermentation aspects of PHA accumulation in cyanobacteria. The biochemical and molecular basis of PHA synthesis in cyanobacteria are not well understood.

The model cyanobacterium *Synechocystis* sp. strain PCC 6803 is considered as a promising candidate for various biotechnological productions because of the availability of its genome sequence information [15] and the ease of genetic manipulation of this strain due to its naturally transformable feature [16]. In this study, *Synechocystis* sp. was metabolically engineered by increasing the flux of intermediates to PHA biosynthesis and introducing a PHA synthase with higher activity. RNA-seq analysis was carried out to examine the differential expression involved in the global biological processes and metabolic pathways during the improved photoautotrophic production of PHA. This information will facilitate the potential use of cyanobacteria for the sustainable production of this ‘green’ polymer.

## Results

### Enhanced PHA Production in Recombinant Cyanobacteria

In the well-studied PHA biosynthetic pathway of *Cupriavidus necator*, P(3HB) synthesis occurs in a three-step reaction and starts with the condensation of acetyl-CoA to acetoacetyl-CoA by β-ketothiolase [17]. Under photosynthetic conditions, it was hypothesized that the acetyl-CoA pool in cyanobacteria is insufficient to drive the thermodynamically unfavorable condensation reaction forward [18]. Instead of relying solely on the native β-ketothiolase-mediated condensation to form acetoacetyl-CoA, an acetoacetyl-CoA synthase (*nphT7_S_*_s_) from *Streptomyces* sp. CL190 was incorporated in the P(3HB) pathway design. The *nphT7_S_*_s_ gene catalyzes the irreversible condensation of acetyl-CoA and malonyl-CoA to give acetoacetyl-CoA, driving the reaction toward the formation of PHA. The evolution of carbon dioxide from the condensation reaction effectively pushes the reaction toward the formation of acetoacetyl-CoA [19]. A highly active PHA synthase from *Chromobacterium* sp. USM2 (*phaC_C_*_s_) [20] was co-expressed with *nphT7_S_*_s_ to improve the photosynthetic production of P(3HB) in *Synechocystis* sp. In view of the stimulatory effects of nutrient limitation, carbon supplementation and air-exchange limitation on PHA accumulation [7], [21], biosynthesis experiments were designed under these cultivation conditions: N- or P-deficient (nitrogen- or phosphorus-deficient), air-exchange limitation, and/or in the presence of carbon sources (CO_2_, acetate and/or fructose).

**Figure 1:**
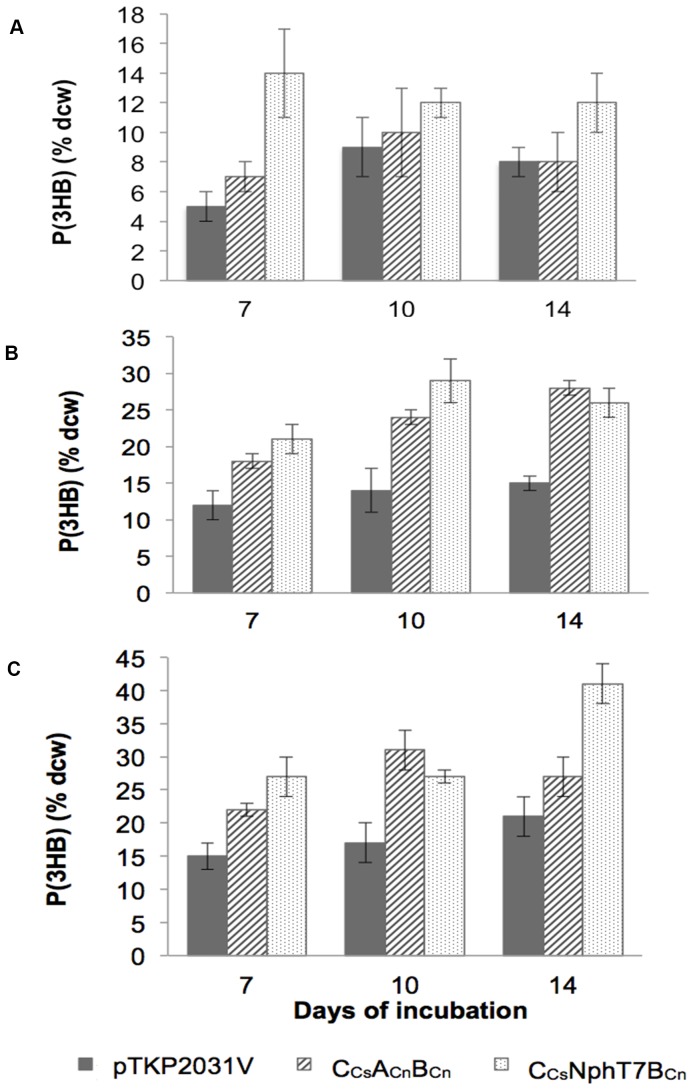
Comparison of P(3HB) accumulation in *Synechocystis* PCC 6803 strains pTKP2031V, C_Cs_A_Cn_B_Cn_ and C_Cs_NphT7B_Cn_. Cells were cultivated on modified BG-11 media, (A) bubbled with 2-3% CO_2_ (B) supplemented with 0.4%(w/v) acetate, incubated with shaking and (C) supplemented with 0.4%(w/v) acetate under air-exchange limiting conditions and incubated with shaking. They were harvested after the specified incubation time (7, 10 or 14 days). Data shown are the means and standard deviation of triplicates.

For the design of PHA production pathway in *Synechocystis* sp., the vector plasmid pTKP2031V was used for the insertion of transgenes into the genome via homologous recombination between sites *slr2030* and *slr2031* [22]. *Synechocystis* sp. was transformed with a plasmid harboring *phaC_C_*_s_, *nphT7_S_*_s_ and *C*. *necator* acetoacetyl-CoA reductase (*phaB_Cn_*) genes under the control of the light-inducible *psbAII* promoter. The successful transformant strain C_Cs_NphT7B_Cn_ was analyzed for PHA production under a two-stage culture system consisting of sequential cell growth and PHA accumulation phases. The strain C_Cs_NphT7B_Cn_ achieved an encouraging direct photosynthetic production of PHA from CO_2_, with a maximum of 14 wt% P(3HB) content on day 7 of cultivation (Fig. 1A). In comparison, strain C_Cs_A_Cn_B_Cn_ expressing *phaC_C_*_s_, *C*. *necator* β-ketothiolase (*phaA_Cn_*) and *phaB_Cn_* recorded a reduction in P(3HB) content (7 wt%) under the same cultivation conditions. The strain pTKP2031V, with only a kanamycin resistance cassette integrated into the genome, showed the lowest P(3HB) production potential (5 wt%). Prolonged incubation until day 14, however, did not exert any significant impact on the P(3HB) accumulation potential of the cyanobacteria under photoautotrophic conditions. At higher cell densities, P(3HB) accumulation may be limited by competition for carbon dioxide and light.

In order to boost P(3HB) production, an exogenous carbon source [0.4%(w/v) acetate] was provided to the cyanobacterial cultures at the PHA accumulation phase. Strain C_Cs_NphT7B_Cn_ recorded the highest PHA content of 29 wt% on day 10 of incubation (Fig. 1B). The increase in the P(3HB) pool resulting from the addition of a carbon source affirms earlier findings on the effect of external carbon source supplementation on PHA production [7], [21]. In the case of air-exchange limitation effect, a significant increase in P(3HB) was observed (up to 41 wt%) for strain C_Cs_NphT7B_Cn_ (Fig. 1C). These observations imply that the P(3HB) accumulation potential of *Synechocystis* sp. is affected by the provision of carbon source and air-exchange. Interestingly, the increase in CO_2_ supply (5%) to photoautotrophic cultures of *Synechocystis* sp. was found to increase the PHA content up to 16 wt% in strain C_Cs_NphT7B_Cn_ (Table 1). The simultaneous addition of acetate and fructose to N- or P-deficient cultures of strain pTKP2031V showed a reduction in P(3HB) accumulation compared to photoautotrophic conditions (5% CO_2_). There were no significant changes in the P(3HB) content of strains C_Cs_NphT7B_Cn_ and C_Cs_A_Cn_B_Cn_ under the same cultivation conditions. The order of PHA-producing potential of the recombinant *Synechocystis* sp. on day 7 of incubation and under the cultivation conditions tested in this study is C_Cs_NphT7B_Cn_> C_Cs_A_Cn_B_Cn_>pTKP2031V.

**Table 1:** P(3HB) accumulation in recombinant *Synechocystis* sp. PCC 6803 under various treatment conditions.

Treatment	P(3HB) (%) w/w of dry cells
pTKP2031V	
N-deficiency, CO_2_ (5%)	10±1
P-deficiency, Acetate, Fructose	3±1
N-deficiency, Acetate, Fructose	6±1
C_Cs_A_Cn_B_Cn_	
N-deficiency, CO_2_ (5%)	10±2
P-deficiency, Acetate, Fructose	8±1
N-deficiency, Acetate, Fructose	12±1
C_Cs_NphT7B_Cn_	
N-deficiency, CO_2_ (5%)	16±4
P-deficiency, Acetate, Fructose	18±3
N-deficiency, Acetate, Fructose	15±2

Comparison of P(3HB) accumulation in *Synechocystis* sp. PCC 6803 strains pTKP2031V, C_Cs_A_Cn_B_Cn_ and C_Cs_NphT7B_Cn_. Cells cultivated on modified BG-11 media under the indicated cultivation conditions were harvested after 7 days of incubation. Data shown are the means and standard deviation of triplicates.

### Expression Levels of PHA Synthesis-related Genes

**Figure 2:**
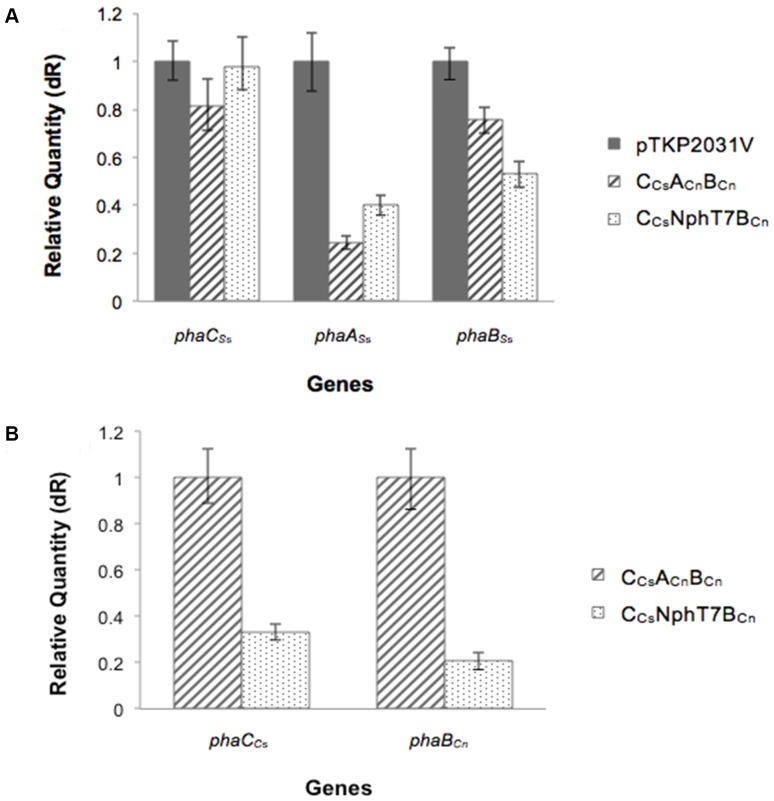
Comparative quantification of expression levels of PHA biosynthetic genes in (A) *Synechocystis* sp. PCC 6803 strains C_Cs_NphT7B_Cn_, C_Cs_A_Cn_B_Cn_ (target) relative to pTKP2031V (calibrator) and (B) C_Cs_NphT7B_Cn_ (target) relative to C_Cs_A_Cn_B_Cn_ (calibrator). Cells were cultivated in modified BG-11 media bubbled with 2-3% CO_2_.

The expression levels of native PHA biosynthetic genes in *Synechocystis* sp. consisting of *phaC_Ss_*, *phaA_Ss_* and *phaB_Ss_* were monitored by real-time PCR analysis (Fig. 2A and 2B). Surprisingly, comparative quantification of *phaA_Ss_* and *phaB_Ss_* expression levels in *Synechocystis* sp. strain C_Cs_NphT7B_Cn_ relative to pTKP2031V show expression that was approximately 2-fold lower. However, there were no significant differences in the expression level of the native *phaC_Ss_* gene in the recombinant *Synechocystis* sp. (pTKP2013V, C_Cs_A_Cn_B_Cn_ and C_Cs_NphT7B_Cn_) investigated. The expression levels of *phaC_C_*_s_ and *phaB_Cn_* that were introduced into the genome on the same operon showed at least 3-fold lower expression in strain C_Cs_NphT7B_Cn_ compared to C_Cs_A_Cn_B_Cn_. Despite higher levels of PHA accumulation in C_Cs_NphT7B_Cn_, the expression levels of most PHA synthesis-related genes in this strain were relatively lower compared to C_Cs_A_Cn_B_Cn_ and pTKP2013V.

### Analysis of *Synechocystis* sp. Transcriptional Response Under Photoautotrophic PHA Accumulation Conditions

To gain insight into PHA accumulation in cyanobacteria, transcriptomes of recombinant *Synechocystis* sp. with different PHA-producing potential were analyzed. RNA-seq libraries were prepared from cells cultivated for 7 days in N-deficient BG-11 under photoautotrophic conditions. Sequencing was performed using the Illumina platform yielding a total of 93-million reads for 6 samples, with an average of 15.5-million reads per sample. Scatter plots between the two biological replicates for each recombinant *Synechocystis* sp. sample show correlation coefficients between 0.96-0.98, indicating the reproducibility of the sequencing data (Fig. S1). The expression levels for each gene were quantified as reads per kilobase of exon model per million mapped reads (RPKM).

The RNA-seq data provide detailed information on the genes that are regulated in response to photoautotrophic PHA accumulation conditions in recombinant *Synechocystis* sp. strains pTKP2013V, C_Cs_A_Cn_B_Cn_ and C_Cs_NphT7B_Cn_. In general, the highly expressed genes in *Synechocystis* sp. were mainly involved in photosynthesis, the electron transport chain, protein metabolic processes and nucleic acid metabolism (Table S1). In particular, the transcript levels of genes involved in photosystem I (*psaB*, *psaA*, *psaF* and *psaL*) and photosystem II (*psbA3*, *psbA2*, *psbX*, *psbY*, *psbU*, *psbK* and *psbD2*) activities were among the most abundant. A comparison between gene expression in the recombinant strains that were more efficient in PHA production (C_Cs_A_Cn_B_Cn_ andC_Cs_NphT7B_Cn_) relative to the reference strain (pTKP2013V) was made (Table 2). The up-regulated genes in strains C_Cs_A_Cn_B_Cn_ and C_Cs_NphT7B_Cn_ significantly enriched photosynthesis, transport and cell communication. In contrast, the down-regulated genes were found to be involved mostly in the metabolism of cofactors and vitamins, protein metabolic processes and DNA-binding. The photosystem I reaction center subunit XII gene (*psaM*) that is detected only in cyanobacteria was strongly up-regulated in both C_Cs_A_Cn_B_Cn_ and C_Cs_NphT7B_Cn_. Another photosystem I reaction center subunit gene, *psaJ* was found to be up-regulated more than 10-fold. Both of these subunits are required to form a functional photosystem I [23]. In addition to genes encoding the photosystem I subunits, photosystem II-associated genes were among the significantly up-regulated genes. PsbX and PsbK, that have been found essential for the stability of photosystem II [24], were induced more than 5-fold in both C_Cs_A_Cn_B_Cn_ andC_Cs_NphT7B_Cn_. Up-regulation of cytochrome B6-f complex subunits, PetG and PetL that are important for either stability or assembly of the complex, was also observed [25]. Two genes involved in porphyrin and chlorophyll metabolism, magnesium-protoporphyrin IX monomethyl ester cyclase (*sll1874*) and protoheme IX farnesyltransferase (*sll1899*) were up-regulated in both strains. Collectively, genes encoding proteins involved in several aspects of photosynthetic activity, e.g. photosystem I and II, cytochrome and chlorophyll metabolism were up-regulated in recombinant *Synechocystis* sp. that were actively synthesizing PHA.

**Table 2:** Genes up-regulated in recombinant *Synechocystis* sp. strains C_Cs_A_Cn_B_Cn_ and C_Cs_NphT7B_Cn_ (compared with pTKP2031V)^a^.

Gene ID	Description	Fold change		Expression level^b^			Functional category
		(C_Cs_A_Cn_B_Cn_ vs pTKP2031V)	(C_Cs_NphT7B_Cn_ vs pTKP2031V)	pTKP2031V	C_Cs_A_Cn_B_Cn_	C_Cs_NphT7B_Cn_	
ssr1169	salt-stress induced hydrophobic peptide	31.93	29.34	22.77	726.97	668.02	cation transport
slr1064	mannosyltransferase	29.17	20.04	7.85	228.9	157.24	polysaccharide metabolic process
smr0005	photosystem I reaction center subunit XII, PsaM	22.83	12.96	22.21	507.15	287.87	photosynthesis
sml0008	photosystem I reaction center subunit IX, PsaJ	17.51	11.21	45.43	795.62	509.14	photosynthesis
sll1161	adenylate cyclase	11.6	10.21	16.04	186.09	163.82	nucleotide metabolic process
slr2114	spore coat polysaccharide biosynthesis protein, SpsC	10.27	9.45	7.83	80.37	73.95	metabolic process
sml0002	photosystem II protein, PsbX	10.71	7.9	186.1	1,992.29	1,469.60	photosynthesis
sml0005	photosystem II reaction center protein K, PsbK	6.98	7.61	106.49	743.15	810.34	photosynthesis
smr0010	cytochrome B6-f complex subunit, PetG	6.96	7.1	175.41	1,220.93	1,245.29	photosynthesis
sll0247	iron-stress chlorophyll-binding protein	4.49	7	61.36	275.41	429.3	photosynthesis
slr0756	circadian rhythm protein	3.21	4.93	56.8	182.13	280.03	circadian rhythm
sll0986	Transposase	3.57	4.68	84.36	301.33	395.05	DNA-binding
slr1318	iron(III) dicitrate ABC transporter permease	3.5	4.18	19.17	67.18	80.06	transport
sll1270	glutamine ABC transporter	2.52	4.15	91.4	230.36	379.34	amino acid transport
sll1405	biopolymer transport protein	2.25	4.02	15.07	33.95	60.53	protein transport
slr1693	PatA subfamily protein	3.2	4.02	55.32	177.23	222.58	intracellular signal transduction
sll1994a	cytochrome B6f complex subunit, PetL	3.92	3.97	17.59	68.91	69.77	energy metabolism
sll0778	ABC transporter	3.39	3.8	14.72	49.95	55.89	lipid transport
slr1760	regulatory components of sensory transduction system	3.33	3.67	23.44	78.05	86.01	signal transduction
sll1874	magnesium-protoporphyrin IX monomethyl ester cyclase	3.19	3.64	21.02	67.16	76.58	porphyrin and chlorophyll metabolism
slr0312	NarL subfamily protein	2.54	3.61	50.47	128.41	182.41	intracellular signal transduction
sll0789	OmpR subfamily protein	1.84	3.58	92.86	170.51	332.85	intracellular signal transduction
slr1755	NAD(P)H-dependent glycerol-3-phosphate dehydrogenase	3.02	3.32	28.65	86.61	95.08	glycerophospholipid metabolism
sll1821	50S ribosomal protein L13	2.71	3.32	86.75	235.5	288.09	translation
slr0611	solanesyl diphosphate synthase	2.52	3.21	36.57	92.07	117.33	metabolic process
sll0792	transcriptional repressor, SmtB	1.92	3.09	77.57	149.25	239.65	DNA-binding
sll0779	PleD protein	2.07	2.99	36.05	74.56	107.94	signal transduction
sll1740	50S ribosomal protein L19	1.6	2.96	356.8	572.54	1,054.92	translation
slr0984	CDP-glucose-4,6-dehydratase	2.7	2.88	20.43	55.12	58.88	amino sugar and nucleotide sugar metabolism
sll0790	sensory transduction histidine kinase	1.48	2.85	98.45	145.37	280.87	signal transduction
slr2079	glutaminase	2.31	2.79	65.16	150.48	182.1	cellular amino acid metabolic process
slr2123	D-isomer specific 2-hydroxyacid dehydrogenase	2.72	2.78	21.83	59.38	60.62	carbohydrate metabolic process
sll0643	urease accessory protein G	1.57	2.73	44.07	68.98	120.44	GTP catabolic process
slr1498	hydrogenase isoenzyme formation protein, HypD	2.12	2.72	33.82	71.78	91.98	protein metabolic process
sll1041	ABC transporter	1.95	2.61	75.65	147.32	197.09	phosphate transport
slr1982	chemotaxis protein, CheY	1.59	2.6	298.23	474.89	774.35	intracellular signal transduction
slr2131	cation or drug efflux system protein	2.29	2.6	26.11	59.75	67.83	transport
slr1595	Na/H antiporter	2.45	2.59	13.76	33.65	35.64	cation transport
slr1912	anti-sigma F factor antagonist	1.87	2.59	75.93	141.71	196.56	regulation of transcription
ssl2296	pterin-4-alpha-carbinolamine dehydratase	1.81	2.51	56.88	103.21	142.56	tetrahydrobiopterin biosynthetic process
sll1428	P3 protein	2.18	2.49	17.28	37.61	43.1	cation transport
sll0080	N-acetyl-gamma-glutamyl-phosphate reductase	1.6	2.48	127.18	204	315.28	amino acid metabolic process
sll1899	protoheme IX farnesyltransferase	1.43	2.47	79.37	113.61	195.82	porphyrin and chlorophyll metabolism
sll1291	PatA subfamily protein	2.03	2.45	89.47	181.58	219.06	signal transduction
slr0889	ABC1-like protein	1.82	2.41	33.73	61.35	81.44	energy metabolism
sll1249	bifunctional pantoate ligase/cytidylate kinase	1.5	2.39	50.72	75.95	121.45	pyrimidine base metabolic process
slr1909	NarL subfamily protein	2.13	2.38	35.46	75.5	84.51	signal transduction
slr1805	sensory transduction histidine kinase	1.34	2.32	80.73	108.44	187.15	signal transduction
sll1229	hybrid sensory kinase	1.78	2.31	49.42	88.21	113.98	signal transduction

^a^ Only the top 50 highest increase in fold-change and genes encoding known proteins are shown.

^b^ The values shown represent the mean of two independent biological replicates.

On the other hand, transcript levels of genes encoding protein metabolism (transcription, translation, amino acid synthesis, etc.) decreased in the recombinant *Synechocystis* sp. strains C_Cs_A_Cn_B_Cn_ and C_Cs_NphT7B_Cn_ (Table S2). The decrease in transcript levels of genes encoding these proteins [DNA mismatch protein (MutL), methionine sulfoxide reductase B (*sll1680*), prohibitin (*slr1106*), exoenzyme S synthesis protein B (ExsB), 3-dehydroquinate dehydratase (AroQ) and hydrogenase (HypA)] may be related to the reduced growth of *Synechocystis* sp. under N-deficient conditions. Cells response to nutrient-limiting conditions by accumulating PHA and at the same time slowing down metabolic activities. Reductions in expression levels of genes related to the metabolism of cofactors and vitamins [lipopeptide antibiotics iturin a biosynthesis protein (*slr0495*), cobalamin synthase (CobS), 4-hydroxythreonine-4-phosphate dehydrogenase (PdxA), cobalt-precorrin-6x reductase (CobK), riboflavin biosynthesis protein (RibG), lipolytransferase (LipB) and o-succinylbenzoate synthase (*sll0409*)] were observed.

A comparison between gene expression in the recombinant *Synechocystis* sp. strains C_Cs_NphT7B_Cn_ and C_Cs_A_Cn_B_Cn_ was made to gain substantial insights into the global responses of cyanobacteria to accommodate the extensive accumulation of PHA (Table 3). The analysis showed that strain C_Cs_NphT7B_Cn_ employed a combination of induced stress response, photosynthesis, energy metabolism and transport during the PHA accumulation phase. Notably, genes encoding proteins involved in several aspects of photosynthetic activity e.g. uroporphyrinogen decarboxylase (HemE), ferredoxin component (*slr1205*), protochlorophilide reductase subunit (BchB), protohome IX farnesyltransferase (CtaB), photosystem II reaction center protein N (PsbN) and iron-stress chlorophyll-binding protein (IsiA) were up-regulated in strain C_Cs_NphT7B_Cn_ compared to C_Cs_A_Cn_B_Cn._ The increased photosynthetic activity suggests that the carbon fixing capacity was enhanced to accommodate the increased diversion of carbon to polymer formation. Polyhydroxyalkanoate are bacterial storage compounds synthesized in response to conditions of physiological stress [26]. In the current study, stress-related genes in cyanobacteria include co-chaperonin (*groES*), Holliday junction resolvase (*ruvC*), molecular chaperon (*groEL*), superoxide dismutase (*sodB*) and heat shock protein 90 (*htpG*) were modestly up-regulated. As it was proposed that PHA accumulation confers survival and stress tolerance in a changing environment [27], stress conditions may trigger responses that favor PHA production. In addition, the transcript level for the global nitrogen regulator, NtcA was detected at an increased level. NtcA is known to regulate the expression of a large number of genes involved in nitrogen metabolism [28] and induction of the gene encoding this protein can be related to the N-deficient cultivation conditions that were applied to increase PHA biosynthesis in *Synechocystis* sp. Conversely, down-regulation of the genes involved in DNA-binding, transport, translation and DNA repair were observed in strain C_Cs_NphT7B_Cn_ (Table S3).

**Table 3:** Genes up-regulated in recombinant *Synechocystis* sp. strain C_Cs_NphT7B_Cn_ (compared with C_Cs_A_Cn_B_Cn_)^a^.

Gene ID	Description	Fold change	Expression level^b^		Functional category
		(C_Cs_NphT7B_Cn_vsC_Cs_A_Cn_B_Cn_)	C_Cs_A_Cn_B_Cn_	C_Cs_NphT7B_Cn_	
slr2075	co-chaperonin, GroES	3.26	328.12	1,069.34	protein folding
slr1204	serine protease, HtrA	2.73	1,046.94	2,860.72	cell communication
slr1316	iron(III) dicitrate ABC transporter permease	2.37	15.33	36.3	iron transport
ssr2595	high light inducible protein	2.19	25.55	55.96	chlorophyll-binding
sll0379	UDP-N-acetylglucosamine acyltransferase	2.14	28.2	60.47	lipopolysaccharide biosynthetic process
sll0789	OmpR subfamily protein	2.13	108.53	231.24	intracellular signal transduction
sll0790	sensory transduction histidine kinase	2.12	90.22	191.02	signal transduction
sll0896	Holliday junction resolvase, RuvC	2.09	17.14	35.89	DNA repair
slr2076	molecular chaperone, GroEL	2.05	733.79	1,507.39	protein folding
slr1279	NADH dehydrogenase subunit A	2.05	25.85	52.92	electron transport chain
slr0536	uroporphyrinogen decarboxylase, HemE	2.02	80.15	162.23	porphyrin and chlorophyll metabolism
slr1675	hydrogenase expression/formation protein, HypA	2.01	111.92	225.29	cellular protein modification process
slr1202	lactose ABC transporter permease	1.96	16.49	32.23	transport
sll1740	50S ribosomal protein L19	1.93	427.21	825.93	translation
sll1538	beta-glucosidase	1.92	21.65	41.64	carbohydrate metabolism
slr1205	ferredoxin component	1.92	214.39	412.13	photosynthesis
ssr2049	protochlorophillide reductase 57 kD subunit, BchB	1.9	30.51	57.98	photosynthesis
slr1805	sensory transduction histidine kinase	1.89	63.65	120.35	signal transduction
sll1899	protoheme IX farnesyltransferase, CtaB	1.88	67.28	126.68	porphyrin and chlorophyll metabolism
slr1256	urease subunit gamma	1.86	38.67	72.03	nitrogen metabolism
sll0109	chorismatemutase	1.84	34.12	62.78	amino acid biosynthetic process
slr1516	superoxide dismutase, SodB	1.83	1,356.38	2,485.21	immune system process
sll1423	global nitrogen regulator, NtcA	1.82	376.21	682.95	transcription
sll0045	sucrose phosphate synthase	1.81	22.41	40.57	starch and sucrose metabolism
sll0899	bifunctional N-acetylglucosamine-1-phosphate uridyltransferase/glucosamine-1-phosphate acetyltransferase	1.8	19.64	35.42	carbohydrate metabolism
slr1147	sensory transduction histidine kinase	1.8	34.66	62.29	signal transduction
ssl2233	30S ribosomal protein S20	1.8	457.05	821.29	translation
smr0009	photosystem II reaction center protein N, PsbN	1.79	113.3	202.33	photosynthesis
slr1728	potassium-transporting ATPase subunit A	1.77	48.53	85.79	ion transport
sll2010	UDP-N-acetylmuramoyl-L-alanyl-D-glutamate synthetase	1.77	17.31	30.59	peptidoglycan biosynthetic process
slr0676	adenylylsulfate kinase	1.76	18.58	32.79	sulfur metabolism
slr1982	chemotaxis protein, CheY	1.76	344.98	605.5	intracellular signal transduction
sll1869	3-chlorobenzoate-3,4-dioxygenase	1.75	27.55	48.23	oxidation reduction process
sll1085	glycerol-3-phosphate dehydrogenase	1.75	34.23	59.74	glycerophospholipid metabolism
ssl2250	glycoprotein	1.74	99.29	172.65	drug and analog sensitivity
sll1957	arsenical resistance operon repressor	1.74	19.37	33.61	transcription
slr2035	gamma-glutamyl kinase	1.73	22.05	38.08	amino acid biosynthetic process
slr1295	iron transport protein	1.72	88.89	153.22	iron transport
sll0792	transcriptional repressor, SmtB	1.71	93.19	159.74	transcription regulation
sll1405	biopolymer transport protein	1.71	19.58	33.47	protein transport
sll1249	bifunctionalpantoate ligase/cytidylate kinase	1.7	42.91	72.84	pantothenate biosynthetic process
sll1283	sporulation protein, SpoIID	1.7	47.99	81.34	sporulation
ssr1386	NADH dehydrogenase subunit, NdhL	1.69	51.61	87.32	energy metabolism
slr1843	glucose-6-phosphate 1-dehydrogenase	1.67	64.14	107.41	carbohydrate metabolism
sll0247	iron-stress chlorophyll-binding protein	1.65	186.8	308.95	photosynthesis
sll1468	beta-carotene hydroxylase	1.65	87.04	143.75	carotenoid biosynthetic process
slr1476	aspartate carbamoyltransferase	1.65	57.68	95.25	pyrimidine biosynthetic process
sll0648	lipophilic protein	1.65	26.01	42.85	lipid transport
sll0923	exopolysaccharide export protein	1.64	181.51	297.86	lipopolysaccharide biosynthetic process
sll0430	heat shock protein 90, HtpG	1.64	186.42	305.82	protein folding

^a^ Only the top 50 highest increase in fold-change and genes encoding known proteins are shown.

^b^ The values shown represent the mean of two independent biological replicates.

## Discussion

Current limitation of direct photosynthetic production using cyanobacteria is the relatively low PHA content obtained. In this study, it was encouraging to obtain 14 wt% of P(3HB) from direct photosynthetic fixing of carbon dioxide without the addition of an external carbon source. Although cyanobacteria have simple nutrient requirements, the addition of 0.4%(w/v) acetate was found to increase P(3HB) content up to 41 wt% under air-exchange limiting conditions. Previous studies suggested that enhanced P(3HB) accumulation was the result of direct metabolism of acetate for PHA synthesis by employing an existing pathway operating in cyanobacteria [7], [21]. The provision of exogenous carbon was found to have a positive impact on PHA accumulation albeit at concentrations that were 10- to 20-fold lower than those required by heterotrophic bacteria. Recently, the development of new photobioreactors for mass cultivation of cyanobacteria is in progress and these findings will greatly aid the use of cyanobacteria for potential industrial applications [29], [30].

Early studies indicate that the PHA biosynthetic genes of *Synechocystis* sp. 6803 do not co-localise together to form an operon [31], [32]. Instead, the PHA synthase of *Synechocystis* sp. consisting of *phaC* and *phaE* subunits are linked in the genome and co-expressed. On the other hand, the β-ketothiolase and acetoacetyl-CoA reductase of *Synechocystis* sp. do not map close to the PHA synthase locus but are probably clustered together and constitute an operon in a different section of the genome. The expression levels of these two genes were surprisingly lower in the recombinant *Synechocystis* sp. strains C_Cs_A_Cn_B_Cn_ and C_Cs_NphT7B_Cn_ that had higher PHA production potential compared to strain pTKP2013V that accumulated a lower content of PHA. These results suggest that the endogenous PHA biosynthetic pathway operating in *Synechocystis* sp. did not have a significant impact on the PHA-synthesizing abilities of strains C_Cs_A_Cn_B_Cn_ and C_Cs_NphT7B_Cn_.

**Figure 3:**
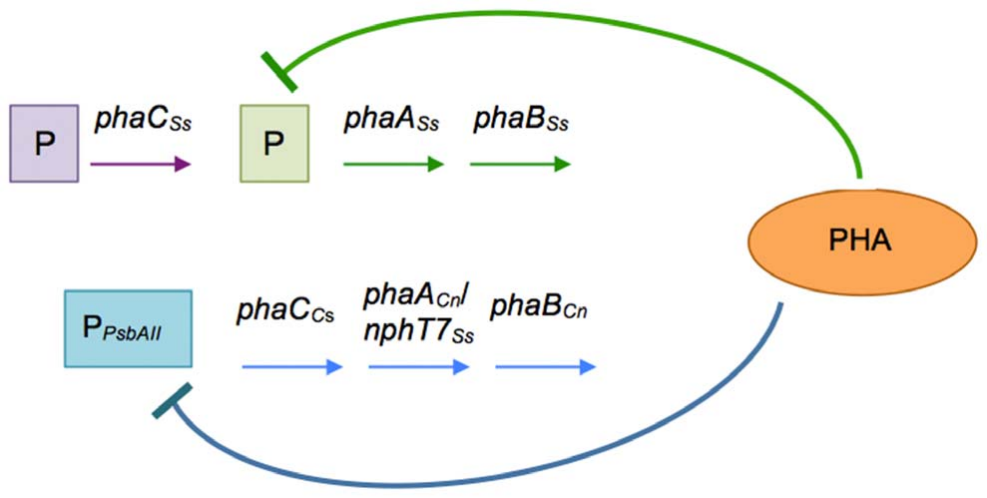
The scheme shows the regulation of PHA synthesis-related gene expression in recombinant *Synechocystis* sp.

The *Chromobacterium* sp. PHA synthase and *C*. *necator* acetoacetyl-CoA reductase that were introduced into the genome as an operon showed similar lower expression in strain C_Cs_NphT7B_Cn_. The observation that the expression levels of most of the PHA biosynthetic genes were lower in strain C_Cs_NphT7B_Cn_ suggests that the concentration of these enzymes is not the limiting factor in achieving higher PHA accumulation. Based on the results presented here, the transcription of genes encoding enzymes involved in PHA biosynthesis is highly regulated and may be affected by the PHA content in the cells (Fig. 3). When the PHA accumulated by the cells has exceeded a certain threshold level, adequate levels of the enzymes may already be present to meet the biosynthetic demand. Thus, the PHA granule itself or some other sensing factors may exert negative feedback on the expression of these enzymes. However, the expression levels of the enzyme catalyzing the last step of PHA biosynthesis, *Synechocystis* sp. PHA synthase, remained grossly constant in all recombinant *Synechocystis* sp. because negative feedback regulations are likely exerted in the upper part of the pathway.

**Figure 4:**
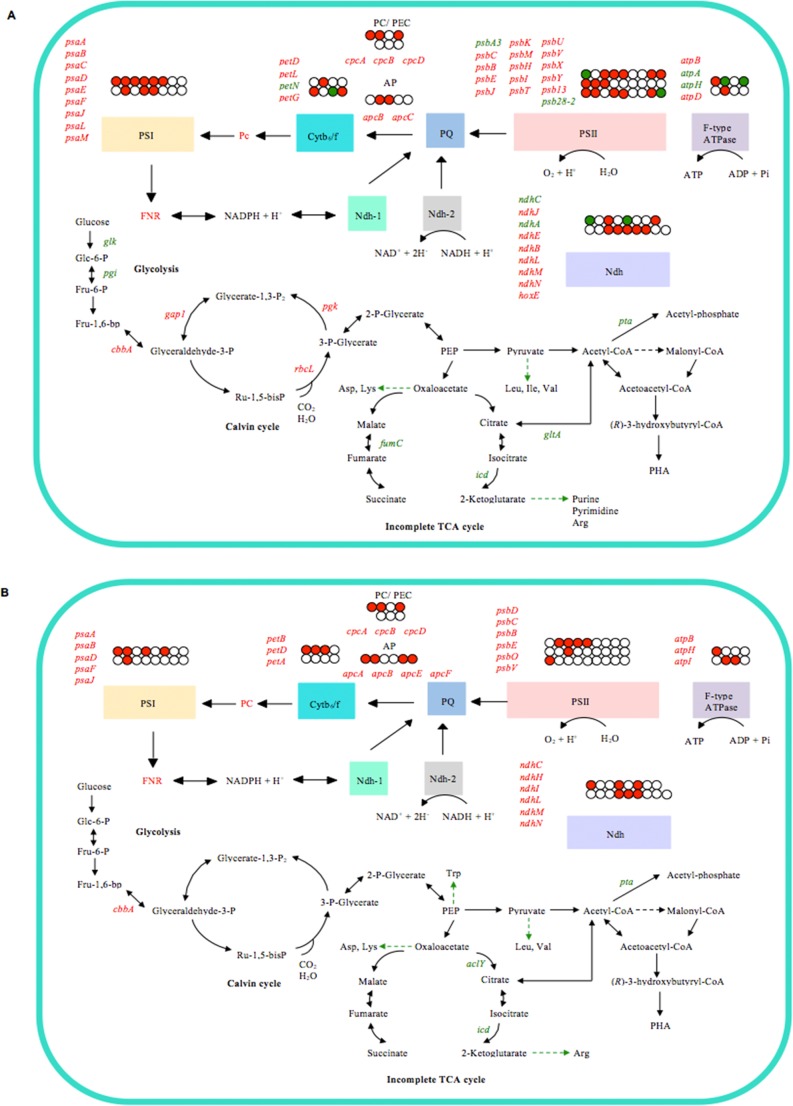
The scheme shows the cellular changes in recombinant *Synechocystis* sp. strains (a) C_Cs_A_Cn_B_Cn_ and C_Cs_NphT7B_Cn_ (compared with pTKP2031V) (b) C_Cs_NphT7B_Cn_ (compared with C_Cs_A_Cn_B_Cn_) under photoautotrophic PHA biosynthesis conditions. Only a selection of cellular changes is shown. The genes or pathways that are up-regulated are in red; the downregulated ones are in green. Black dashed lines indicate the engineered route. AP, allophycocyanin; PC/PEC, phycocyanin/phycoerythrocyanin; Cytb_6_/f, cytochrome b6/f complex; PQ, plastoquinone; FNR, ferredoxin-NADP(+) reductase; Pc, plastocyanin; PSI, photosystem I; PSII, photosystem II; Ndh, NADH dehydrogenase; Glc-6-P, glucose-6-phosphate; Fru-6-P, fructose-6-phosphate; Fru-1,6-bp; fructose-1,6-biphosphate; Glycerate-1,3-P_2_, 1,3-biphosphoglycerate; 3-P-Glycerate, 3-phosphoglycerate; Ru-1,5-bisP, ribulose-1,5-biphosphate; PEP, phosphoenolpyruvate.

Previous genetic studies have focused on the engineering of various bacteria or plant hosts for PHA production, but less is known about the global transcriptional changes of the recombinant host under a PHA-synthesizing environment. A comprehensive view of the cyanobacterial transcriptome during cultivation under conditions favorable for PHA synthesis was generated using RNA-seq analysis. One particularly interesting observation is the up-regulation of photosynthetic activity in recombinants *Synechocystis* sp. with higher PHA-synthesizing potential (Fig. 4 A and B). In recent years, there has been tremendous interest in strategies to improve photosynthetic activity in crops [33], [34]. It has been suggested that an increase in photosynthetic activity will improve the yield of crops and provide a potential solution to future food shortages [35]. In this context, the increase of photosynthetic activity in cyanobacteria may explain the higher PHA accumulation observed in recombinant *Synechocystis* sp. strains C_Cs_NphT7B_Cn_ and C_Cs_A_Cn_B_Cn_.

The gene encoding one of the most important enzymes in carbon fixation, the ribulose-1,5 biphosphate carboxylase/oxygenase (RuBisCo) large subunit (*rbcL*), was up-regulated in both C_Cs_NphT7B_Cn_ and C_Cs_A_Cn_B_Cn_. RuBisCo is a biologically important enzyme that catalyzes the first step of the reaction that converts atmospheric carbon dioxide into organic carbon [36]. Besides RuBisCo, genes encoding proteins involved in different aspects of photosynthesis and electron transport chain were significantly induced in both C_Cs_NphT7B_Cn_ and C_Cs_A_Cn_B_Cn._ In particular, the induction of photosynthesis and electron transport chain-related genes was most prominent in strain C_Cs_NphT7B_Cn_ with the highest PHA accumulation, suggesting the possible correlation of photosynthetic activity with PHA content. The *Synechocystis* sp. cells may utilize enhanced photosynthesis, carbon fixation and electron transport chain activities as a means to provide precursors that are necessary to drive the production of PHA. The increased photosynthetic production of PHA reveals that similar metabolic engineering approaches can be applied to the production of biofuels or chemicals using this versatile organism. As cyanobacteria and plants share similar photosynthetic machinery, it is likely that the strategy can be extended in future efforts to improve PHA production in higher plants.

In living cells, catabolic reactions that produce energy and anabolic biosynthetic reactions are regulated to maintain a balance of supply and demand. To cope with the higher PHA production demand, carbon dioxide fixing was enhanced to replenish the pool of carbon that was lost to PHA formation. Concomitant with the increase in photosynthetic activity, the flow of newly fixed carbon dioxide into biosynthetic reactions other than PHA was reduced. Genes encoding metabolism of cofactors and vitamins as well as protein metabolic process were found to be down-regulated in strains C_Cs_NphT7B_Cn_ and C_Cs_A_Cn_B_Cn._ The reduced growth of recombinant *Synechocystis* sp. under nutrient-deficient cultivation conditions may account for the depression of these metabolic processes. These cellular anabolic reactions were regulated to maintain the balance of resources in cells. The expression levels of genes involved in the tricarboxylic acid cycle (TCA) were shown to be down-regulated in strains C_Cs_NphT7B_Cn_ and C_Cs_A_Cn_B_Cn_. These observations agree well with previous finding that reported on the repressed of the TCA cycle genes in *C*. *necator* H16 during PHA production [37].

RNA-seq transcriptome analysis reveals that the heterologous expression of PHA synthesis-related genes in *Synechocystis* sp. affect not only the regulation of PHA biosynthesis but also the preceding pathways that are involved in the provision of precursors for this biosynthesis. The direct photosynthetic production of 14 wt% of P(3HB) from strain C_Cs_NphT7B_Cn_ is the highest value achieved for *Synechocystis* sp. 6803 so far. This work suggests the use of carbon flux as a possible driving force for the biosynthesis of intracellular inclusions e.g. PHA. Future work can be done to confirm this finding by enhancing carbon fixation in cyanobacteria through engineering or overexpressing the enzymes involved in the process.

## Materials and Methods

### Chemicals and Reagents

All chemicals were purchased from Nacalai Tesque (Tokyo, Japan) or Wako Pure Chemical (Tokyo, Japan) unless otherwise specified. KOD Plus high-fidelity DNA polymerase was purchased from Toyobo (Tokyo, Japan). Restriction enzymes and the DNA ligation kits used were from Takara (Shiga, Japan).

### Organism and Culture Conditions

All *Synechocystis* sp. PCC6803 strains (Table S4) were cultivated at 30°C in BG-11 medium [38] buffered with 20 mM HEPES-KOH, pH 8.0, under continuous illumination of 100 µmol photons m^−2^ s^−1^. Liquid cultures were incubated with shaking (100 r.p.m.) or bubbled with air enriched with 2-3% (v/v) CO_2_. *Escherichia coli* DH5α used for plasmid cloning was grown with shaking (180 r.p.m) at 37°C in Lysogeny broth. For the selection and maintenance of plasmids, kanamycin (50 µg/mL) or ampicillin (100 µg/mL) were added. To promote PHA biosynthesis in cyanobacteria, a two-stage cultivation was performed. The cultures were first grown in BG-11 medium until the late exponential phase and then harvested, washed and transferred to BG-11 medium devoid of sodium nitrate. P-deficiency was achieved by cultivating cells in BG-11 medium without K_2_HPO_4_. Different carbon sources [0.2% (w/v) and 0.4% (w/v) of fructose and/or acetate] were added to study the effects of carbon supplementation on PHA accumulation. Air-exchange limiting conditions on cultures were imposed by sealing the mouth of culture vessels with cotton plugs and covering with aluminium foil [7]. The cyanobacterial cultures were cultivated in the above culture conditions for 7, 10 or 14 days, harvested by centrifugation (8000 *g*, 10 min) and then lyophilized.

### Plasmid Construction and Transformation of *Synechocystis* sp

The constructs used for transformation of *Synechocystis* sp. were derived from pTKP2031V (Table S4). pTKP2031V was designed for insertion into the genome via homologous recombination between sites *slr2030* and *slr2031* together with a kanamycin resistance cassette [22]. The expression of all cyanobacterial constructs was under the *psbAII* promoter. The gene cluster containing β-ketothiolase (*phaA_Cn_*) and acetoacetyl-CoA reductase (*phaB_Cn_*) were amplified from chromosomal DNA of *C*. *necator* H16 using primers *phaAB_Cn_* (F; *Nde*I) and *phaAB_Cn_* (R; *Hpa*I) (Table S5). The resulting PCR product was digested with *Nde*I and *Hpa*I and inserted into *Nde*I- and *Hpa*I-digested pTKP2031V to obtain pTKP2031V-*phaAB_Cn_*. The PHA synthase (*phaC_C_*_s_) was prepared from chromosomal DNA of *Chromobacterium* sp. USM2 using primers *phaC_C_*_s_ (F; *Sfu*I) and *phaC_C_*_s_ (R; *Aat*I). This PCR fragment was digested with *Sfu*I and *Aat*I and subcloned into the appropriate restriction sites of pTKP2031V-*phaAB_Cn_* by ligation to yield pTKP2031V-*phaC_C_*_s_ *A_Cn_B_Cn_*. pTKP2031V-*phaC_C_*_s_ *nphT7phaB_Cn_* was constructed by replacing *phaA_Cn_* in pTKP2031V-*phaC_C_*_s_ *A_Cn_B_Cn_* with *nphT7*. The *nphT7* gene from *Streptomyces* sp. CL190 was amplified from a previously prepared template, pHis_*nphT7* [19] using primers *nphT7* (F; *Sfu*I) and *nphT7* (R; *Aat*I). Transformation of *Synechocystis* sp. was performed as described previously [39]. Briefly, 100-200 µL of an exponentially growing culture were mixed with a plasmid solution to a final concentration of 1-2.5 µg/mL. The mixture was then spread onto a nitrocellulose membrane filter placed on a BG-11 plate and incubated overnight (∼12 h) at 30°C under continuous white light (75-100 µmol photons/m^2^s). The membrane filter was transferred onto a new BG-11 plate containing 50 µg/mL kanamycin. Kanamycin-resistant colonies were isolated and replated three times. The presence and complete segregation of the transgene in the cyanobacterial genome were confirmed by PCR analysis and sequencing.

### Quantitative Analysis of PHA

Approximately 25-30 mg of lyophilized cyanobacterial cells were washed with methanol and dried at 65°C overnight. The dry cells were subsequently extracted with chloroform at 65°C for 48 h. The chloroform extract was subjected to methanolysis with a solution consisting of 85%(v/v) methanol and 15%(v/v) concentrated sulphuric acid at 100°C for 140 min [40]. The organic phase comprising the hydroxyacyl methyl esters was analyzed by gas chromatography-mass spectrometry (GC-MS) using the Agilent 7890A GC/5975 MSD system equipped with a HP−5 column (Agilent, USA).

### RNA Preparation

Total RNA was extracted from cells using Trizol reagent (Invitrogen, USA) in combination with the PureLink RNA Mini Kit (Invitrogen, USA) according to manufacturer’s protocol. Any traces of DNA remaining in the RNA samples were removed by digestion with DNase I (Takara, Japan). The quality and quantity of the RNA samples were analyzed using a Bioanalyzer 2100 (Agilent, USA).

### Real-time PCR Analysis

cDNA synthesis was performed with 250 ng of RNA using the QuantiTect Reverse Transcription Kit (Qiagen, USA). Real-time PCR quantification was performed using Thunderbird SYBR qPCR Mix (Toyobo, Japan) and gene-specific primers with the Mx3000P QPCR system (Agilent, USA). The cycling conditions were as follows: 95°C for 10 min, 40 cycles: 95°C for 15 s and 60°C for 1 min. A melting curve analysis (60°C-95°C) was performed after each amplification to ensure specificity of the reaction. Transcript levels were quantified based on determination of the quantification cycle (Ct). The transcript levels of genes of interest were normalized to the level of the housekeeping gene (16S rRNA) used in this study. Comparative quantification was used to compare the expression levels of genes of interest in *Synechocystis* sp. PCC 6803 strains C_Cs_NphT7B_Cn_ and C_Cs_A_Cn_B_Cn_ (target) relative to pTKP2031V (calibrator) and C_Cs_NphT7B_Cn_ (target) relative to C_Cs_A_Cn_B_Cn_ (calibrator).

### RNA-seq Library Preparation, Illumina Sequencing and Data Analysis

For each sample, 2 µg total RNA was subjected to ribosomal RNA depletion using the Ribo-Zero rRNA removal kit (Epicentre, USA). The cDNA libraries for RNA-seq were constructed from total RNA depleted of rRNA using the Illumina TruSeq Stranded mRNA Sample Preparation Kit (Illumina, USA) according to the manufacturer’s specifications. In brief, preparation of the cDNA libraries included the following steps: RNA fragmentation, cDNA synthesis, 3′ ends adenylation, adapter ligation and cDNA template enrichment. Quantification of the libraries was carried out using a Bioanalyzer 2100 (Agilent, USA) and 4 to 6 pM of the template was used for cluster generation. Libraries were sequenced on a Miseq (Illumina, USA) instrument using the 2×250 paired end protocol. The sequence data has been submitted to the NCBI Gene Expression Omnibus (GEO) under accession number GSE50688. The RNA-seq data analysis was performed using CLC Genomics Workbench 6 software (CLC bio, Denmark). Sequence reads were pre-processed to trim low-quality reads and filter reads shorter than 20 bp. The qualified sequence reads were mapped to the *Synechocystis* sp. PCC 6803 genome (NC_000911), allowing a maximum of two mismatches. The reference genome sequences and annotations were downloaded from NCBI (downloaded on May 23, 2013). Sequence reads that mapped to non-coding RNA and reads that did not map to unique positions were excluded from further analysis. The transcript levels were expressed as reads per kilobase of exon model per million mapped reads in which the read count for a gene was normalized by the length of the gene and the total number of reads mapped in the sample [41]. Statistical analysis was performed and genes with a False Discovery Rate (FDR) *p*-value correction <0.05 were determined as differentially regulated genes [42].

## Supporting Information

Figure S1 **Correlation of RNA-Seq data between biological replicates.** Normalized expression values from each sample were used. Correlation coefficients are indicated inside the plots.
(TIF)

Table S1 **Highly expressed genes based on normalized expression level (RPKM values)^a^.**
(DOCX)

Table S2 **Genes down-regulated in recombinant** ***Synechocystis*** **sp. strain C_Cs_A_Cn_B_Cn_ and C_Cs_NphT7B_Cn_ (compared with pTKP2031V)^a^.**
(DOCX)

Table S3 **Genes down-regulated in recombinant** ***Synechocystis*** **sp. strain C_Cs_NphT7B_Cn_ (compared with C_Cs_A_Cn_B_Cn_)^a^.**
(DOCX)

Table S4 **Strains and plasmids used in this study.**
(DOCX)

Table S5 **Primers used in this study.**
(DOCX)
