# Supplementary figures and images for: RNA-Seq Analysis Provides Insights for Understanding Photoautotrophic Polyhydroxyalkanoate Production in Recombinant Synechocystis Sp
Source: PLoS One. 2014 Jan 22;9(1):e86368. doi: 10.1371/journal.pone.0086368 (PMC3899235; doi:10.1371/journal.pone.0086368)

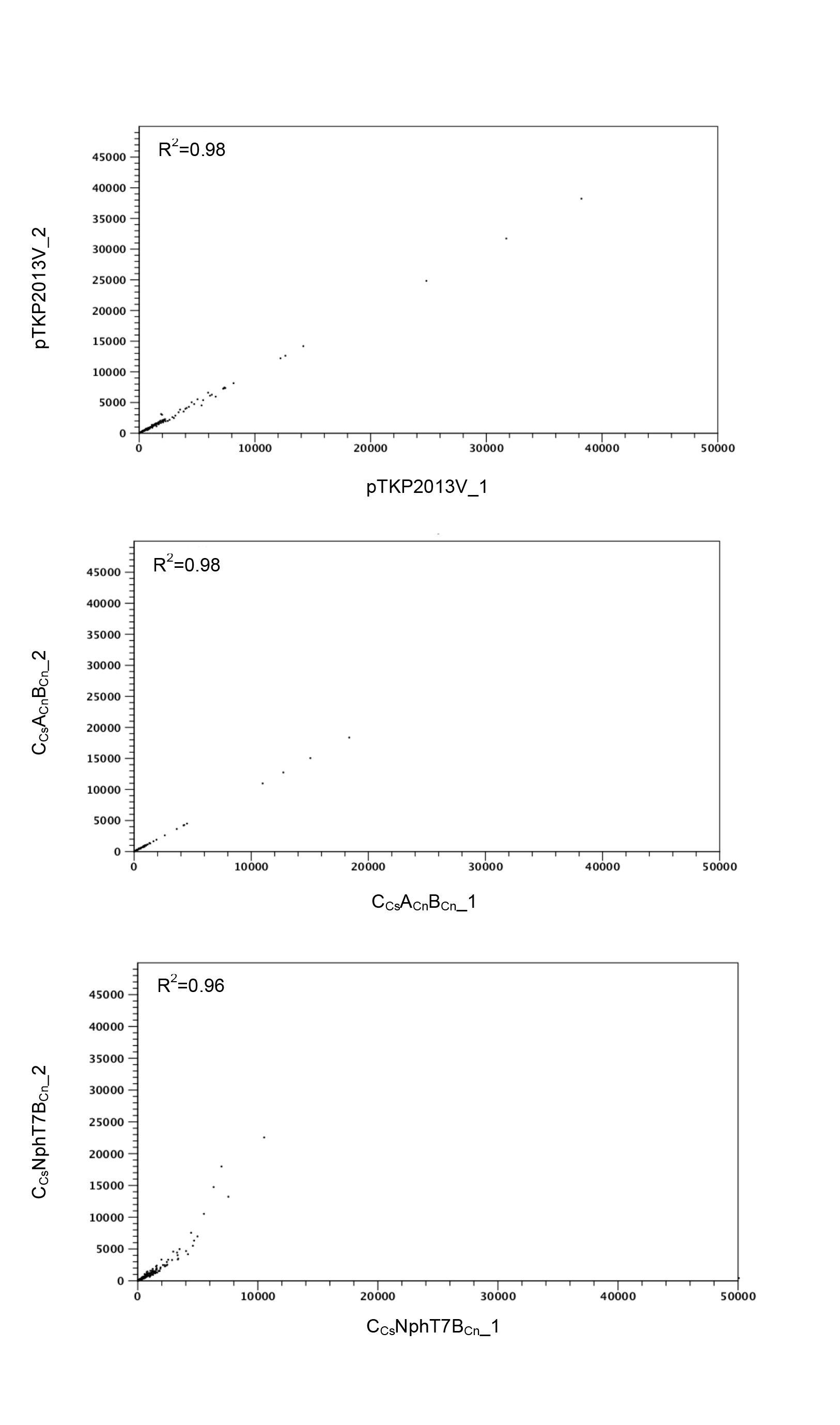

Supplement: Figure S1 — Correlation of RNA-Seq data between biological replicates. Normalized expression values from each sample were used. Correlation coefficients are indicated inside the plots. (TIF) [file pone.0086368.s001.tif]
