# Supplementary material for: RNA-Seq Analysis Provides Insights for Understanding Photoautotrophic Polyhydroxyalkanoate Production in Recombinant Synechocystis Sp
Source: PLoS One. 2014 Jan 22;9(1):e86368. doi: 10.1371/journal.pone.0086368 (PMC3899235; doi:10.1371/journal.pone.0086368)
Supplement: Table S1 — Highly expressed genes based on normalized expression level (RPKM values)a. (DOCX) [file pone.0086368.s002.docx]

**Table S1 Highly expressed genes based on normalized expression level (RPKM values)^a^**

| Gene ID | Description | Expression level^b^ | | | Functional category |
| --- | --- | --- | --- | --- | --- |
|  |  | pTKP2031V | C_Cs_A_Cn_B_Cn_ | C_Cs_NphT7B_Cn_ |  |
| slr0915 | endonuclease | 52,642.10 | 10,546.90 | 33,862.87 | DNA repair |
| sll1514 | chaperone | 17,847.94 | 6,172.01 | 29,815.59 | protein folding |
| ssl1633 | high light inducible protein | 4,442.48 | 3,097.88 | 6,191.88 | chlorophyll-binding |
| sll0517 | RNA binding protein | 2,871.69 | 5,736.29 | 5,075.90 | nucleic acid metabolic process |
| sll1867 | photosystem II D1 protein, PsbA3 | 47,751.89 | 4,152.24 | 4,944.26 | photosynthesis |
| slr1311 | photosystem II D1 protein, PsbA2 | 3,097.88 | 6,393.03 | 4,442.48 | photosynthesis |
| slr1204 | serine protease, HtrA | 1,661.86 | 1,338.70 | 3,767.28 | cell communication |
| slr1516 | superoxide dismutase | 4,442.48 | 1,645.23 | 3,217.80 | immune system process |
| slr0374 | cell division cycle protein | 3,175.86 | 4,911.49 | 3,064.63 | cell division |
| ssr1480 | RNA-binding protein | 1,536.42 | 3,382.32 | 2,210.26 | nucleic acid metabolic process |
| slr2076 | molecular chaperone, GroEL | 832.93 | 931.66 | 1,856.15 | protein folding |
| ssl0601 | 30S ribosomal protein S21 | 1,768.31 | 2,210.26 | 1,724.72 | translation |
| sml0002 | photosystem II protein, PsbX | 186.1 | 1,992.29 | 1,469.60 | photosynthesis |
| ssr1604 | 50S ribosomal protein L28 | 380.48 | 1,565.70 | 1,376.79 | translation |
| ssr2799 | 50S ribosomal protein L27 | 446.85 | 1,387.00 | 1,363.26 | translation |
| slr2075 | co-chaperonin, GroES | 1,197.15 | 454.76 | 1,359.73 | protein folding |
| sll0928 | allophycocyanin-B | 1,361.79 | 1,203.06 | 1,315.99 | electron transport chain |
| [smr0010](http://www.kazusa.ord.jp/cyano/Synechocystis/cgi-bin/geinfo.cgi?type=orf&name=smr0010) | cytochrome B6-f complex subunit, PetG | 175.41 | 1,220.93 | 1,245.29 | photosynthesis |
| [ssl1426](http://www.kazusa.ord.jp/cyano/Synechocystis/cgi-bin/geinfo.cgi?type=orf&name=ssl1426) | 50S ribosomal protein L35 | 732.09 | 1,132.77 | 1,212.94 | translation |
| ssl0707 | nitrogen regulatory protein P-II | 637.85 | 1,375.50 | 1,194.38 | nitrogen compound metabolic process |
| sll1578 | phycocyanin a subunit | 314.81 | 2,809.31 | 1,130.51 | electron transport chain |
| sll1951 | hemolysin | 469.94 | 1,282.77 | 1,074.28 | secondary metabolites biosynthesis, transport and catabolism |
| sll1626 | SOS function regulatory protein | 197.65 | 1,373.58 | 1,060.55 | DNA repair |
| ssl2233 | 30S ribosomal protein S20 | 691.66 | 604.8 | 1,056.53 | translation |
| sll1740 | 50S ribosomal protein L19 | 356.8 | 572.54 | 1,054.92 | translation |
| sml0007 | photosystem II protein, PsbY | 203.44 | 1,040.65 | 1,017.36 | photosynthesis |
| sll1194 | photosystem II complex extrinsic protein U, PsbU | 269.3 | 942.73 | 973.17 | photosynthesis |
| slr1042 | chemotaxis protein, CheY | 939.09 | 823.4 | 924.83 | signal transduction |
| slr0012 | ribulose bisphosphate carboxylase small subunit | 243.26 | 1,100.96 | 892.59 | carbon fixation |
| sll1423 | global nitrogen regulator | 572.7 | 512.69 | 864.69 | transcription |
| slr0604 | GTP-binding protein, LepA | 322.95 | 986.3 | 844.47 | protein biosynthesis |
| sml0005 | photosystem II reaction center protein K, PsbK | 106.49 | 743.15 | 810.34 | photosynthesis |
| slr1835 | photosystem I P700 chlorophyll a apoprotein A2, PsaB | 233.42 | 1,954.39 | 806.7 | photosynthesis |
| slr1982 | chemotaxis protein, CheY | 298.23 | 474.89 | 774.35 | intracellular signal transduction |
| sll0767 | 50S ribosomal protein L20 | 317.16 | 816.23 | 771.31 | translation |
| ssr3307 | preprotein translocase subunit, SecG | 278.46 | 580.02 | 756.51 | protein secretion |
| sll1577 | phycocyanin subunit B | 246.23 | 1,428.72 | 732.09 | electron transport |
| slr0927 | photosystem II D2 protein, PsbD2 | 1,349.39 | 1,914.53 | 708.09 | photosynthesis |
| slr1834 | photosystem I P700 chlorophyll a apoprotein A1, PsaA | 179.97 | 1,713.18 | 693.28 | photosynthesis |
| slr2062 | transposase | 116.39 | 521.05 | 677.7 | DNA-binding |
| ssr1169 | salt-stress induced hydrophobic peptide | 22.77 | 726.97 | 668.02 | cation transport |
| slr0628 | 30S ribosomal protein S14 | 1,039.17 | 888.09 | 645.19 | translation |
| sll1819 | 50S ribosomal protein L17 | 188.12 | 463.8 | 635.21 | translation |
| sll0819 | photosystem I subunit III, PsaF | 193.8 | 1,318.17 | 622.5 | photosynthesis |
| sll0018 | fructose-1,6-bisphosphate aldolase | 250.12 | 808.64 | 618.24 | glycolysis |
| sll1816 | 30S ribosomal protein S13 | 442.32 | 422.68 | 615.49 | translation |
| slr1655 | photosystem I reaction center protein subunit XI, PsaL | 145.85 | 705.77 | 584.44 | photosynthesis |
| sll0416 | chaperonin 2 | 1,289.03 | 391.21 | 567.69 | protein folding |
| sll0020 | ATP-dependent Clp protease regulatory subunit | 708.05 | 873.78 | 561.29 | protein folding |
| slr1205 | ferredoxin component | 256.49 | 311.06 | 557.36 | electron transport chain |

^a^Only the top 50 expressed genes annotated with functions are shown.

^b^The values shown represent the mean of two independent biological replicates.
