# Supplementary material for: RNA-Seq Analysis Provides Insights for Understanding Photoautotrophic Polyhydroxyalkanoate Production in Recombinant Synechocystis Sp
Source: PLoS One. 2014 Jan 22;9(1):e86368. doi: 10.1371/journal.pone.0086368 (PMC3899235; doi:10.1371/journal.pone.0086368)
Supplement: Table S2 — Genes down-regulated in recombinant Synechocystis sp. strain CCsACnBCn and CCsNphT7BCn (compared with pTKP2031V)a. (DOCX) [file pone.0086368.s003.docx]

**Table S2 Genes down-regulated in recombinant *Synechocystis* sp. strain C_Cs_A_Cn_B_Cn_ and C_Cs_NphT7B_Cn_ (compared with pTKP2031V)^a^**

| Gene ID | Description | Fold change | | Expression level^b^ | | | Functional category |
| --- | --- | --- | --- | --- | --- | --- | --- |
|  |  | (C_Cs_A_Cn_B_Cn_  vs pTKP2031V) | (C_Cs_NphT7B_Cn_  vs  pTKP2031V) | pTKP2031V | C_Cs_A_Cn_B_Cn_ | C_Cs_NphT7B_Cn_ |  |
| slr2031 | sigma factor SibG regulation protein, RsbU | -3.26 | -14.09 | 530.79 | 162.95 | 37.67 | metabolic process |
| ssl0296 | transposase | -9 | -12.35 | 97.11 | 10.79 | 7.86 | DNA-binding |
| ssr2595 | high light inducible protein | -24.81 | -11.9 | 1,148.40 | 46.29 | 96.47 | chlorophyll-binding |
| sll1867 | photosystem II D1 protein | -11.5 | -9.66 | 47,751.89 | 4,152.24 | 4,944.26 | photosynthesis |
| slr1543 | DNA-damage-inducible protein | -14.08 | -7.75 | 194.17 | 13.79 | 25.04 | cation transport |
| sll1823 | adenylosuccinate synthetase | -7.25 | -7.6 | 1,220.30 | 168.42 | 160.6 | nucleotide metabolism |
| ssr2898 | transposase | -5.33 | -7.14 | 32.38 | 6.08 | 4.53 | DNA-binding |
| slr0495 | lipopeptide antibiotics iturin a biosynthesis protein | -8.33 | -6.95 | 94.71 | 11.37 | 13.64 | pantothenate and CoA biosynthesis |
| slr1875 | exopolysaccharide synthesis protein, ExoD | -3.42 | -6.7 | 107.32 | 31.38 | 16.01 | cellular component |
| sll1077 | agmatine ureohydrolase | -4.27 | -6.54 | 204.94 | 48.03 | 31.35 | cellular amino acid catabolic process |
| sll1599 | Mn transporter, MntA | -5.54 | -6.07 | 83.46 | 15.07 | 13.74 | transport |
| slr0974 | translation initiation factor IF-3 | -7.57 | -5.7 | 1,327.23 | 175.26 | 232.99 | translation |
| slr0676 | adenylylsulfate kinase | -9.94 | -5.36 | 317.52 | 31.94 | 59.26 | sulfur metabolism |
| slr0636 | cobalamin synthase, CobS | -6.28 | -5.29 | 40.64 | 6.47 | 7.69 | cobalamin biosynthetic process |
| sll0679 | SphX protein | -6.53 | -5.24 | 262.38 | 40.2 | 50.06 | peptide/ nickel transport |
| sll0312 | binding protein-dependent transport system protein, OppB | -5.76 | -5.23 | 318.11 | 55.25 | 60.77 | transport |
| slr1199 | DNA mismatch repair protein, MutL | -5.36 | -5.16 | 83.74 | 15.63 | 16.22 | DNA repair |
| slr0008 | carboxyl-terminal processing protease | -6.1 | -5.13 | 958.05 | 156.98 | 186.86 | pyridoxine biosynthetic process |
| sll0660 | 4-hydroxythreonine-4-phosphate dehydrogenase, PdxA | -6.17 | -4.95 | 107.75 | 17.45 | 21.79 | cofactor biosynthetic process |
| slr0053 | metalloprotease | -2.85 | -4.72 | 158.07 | 55.42 | 33.5 | rRNA processing |
| slr0081 | OmpR subfamily protein | -4.23 | -4.71 | 514 | 121.45 | 109.18 | signal transduction |
| sll1680 | methioninesulfoxide reductase B | -4.39 | -4.69 | 1,032.09 | 235.14 | 220.11 | protein modification process |
| sll1544 | response regulator like-protein | -6.15 | -4.68 | 134.9 | 21.92 | 28.83 | signal transduction |
| slr0252 | cobalt-precorrin-6x reductase, CobK | -3.16 | -4.66 | 145.04 | 45.95 | 31.15 | cobalamin biosynthetic process |
| slr1106 | prohibitin | -7.53 | -4.63 | 103.79 | 13.79 | 22.4 | DNA replication |
| sll0687 | RNA polymerase sigma factor | -3 | -4.62 | 44.45 | 14.84 | 9.62 | transcription regulation |
| slr0352 | transposase | 2.06 | -4.6 | 50.15 | 103.46 | 10.91 | DNA-binding |
| sll1598 | Mn transporter, MntC | -4.33 | -4.45 | 157.64 | 36.43 | 35.45 | metal ion transport |
| slr0018 | fumaratehydratase | -4.68 | -4.31 | 54.1 | 11.56 | 12.56 | fumarate metabolic process |
| sll0271 | transcription antitermination protein, NusB | -6.96 | -4.25 | 123.62 | 17.76 | 29.12 | transcription |
| slr0838 | phosphoribosylaminoimidazole synthetase | -5.28 | -4.23 | 87.23 | 16.51 | 20.62 | purine biosynthetic process |
| slr0066 | riboflavin biosynthesis protein, RibG | -5.1 | -4.18 | 55.45 | 10.87 | 13.27 | riboflavin biosynthetic process |
| slr0853 | ribosomal-protein-alanine acetyltransferase | -5.87 | -4.05 | 679.56 | 115.78 | 167.67 | protein modification process |
| slr0527 | exoenzyme S synthesis protein B, ExsB | -2.74 | -4.03 | 66.17 | 24.14 | 16.41 | folate biosynthetic process |
| slr0994 | lipoyltransferase, LipB | -5.08 | -4.03 | 508.03 | 100 | 126.06 | lipoid acid metabolism |
| sll1112 | 3-dehydroquinate dehydratase, AroQ | -5.1 | -4.01 | 604.8 | 118.57 | 150.66 | amino acid metabolic process |
| sll0409 | O-succinylbenzoate synthase | -5.8 | -4.01 | 60.63 | 10.45 | 15.12 | menaquinone biosynthetic process |
| sll0467 | S-adenosylmethionine--tRNAribosyltransferase-isomerase | -3.32 | -4 | 81.55 | 24.58 | 20.4 | queuosine biosynthetic process |
| sll0738 | molybdate-binding periplasmic protein | -3.58 | -3.91 | 30.77 | 8.59 | 7.88 | molybdate transport |
| sll0537 | ammonium/methylammonium permease | -4.13 | -3.86 | 54.41 | 13.19 | 14.08 | cation transport |
| ssl0769 | transposase | -3.52 | -3.85 | 71.8 | 20.4 | 18.66 | DNA-binding |
| slr1454 | sulfate ABC transporter permease | -2.06 | -3.81 | 29.57 | 14.34 | 7.77 | sulfate transport |
| sll1968 | PmgA protein | -5.78 | -3.79 | 1,525.72 | 263.83 | 402.26 | ATP-binding |
| slr0963 | sulfite reductase subunit beta | -3.37 | -3.73 | 334.68 | 99.2 | 89.74 | sulfur metabolism |
| ssl1922 | transposase | -16.9 | -3.73 | 31.78 | 1.88 | 8.52 | DNA-binding |
| sll1483 | transforming growth factor induced protein | -2.96 | -3.68 | 330.61 | 111.62 | 89.9 | developmental process |
| sll0005 | ABC1-like protein | -3.58 | -3.66 | 277.67 | 77.47 | 75.96 | energy metabolism |
| slr1675 | hydrogenase, HypA | -6.52 | -3.53 | 1,141.36 | 175.18 | 323.4 | protein modification process |
| slr1886 | iojap protein | -2.87 | -3.51 | 574.21 | 200.3 | 163.65 | translation regulation |
| slr0940 | zeta-carotene desaturase | -4.39 | -3.49 | 85.75 | 19.52 | 24.58 | carotenoid biosynthetic process |

^a^Only the top 50 highest decrease in fold-change and genes encoding known proteins are shown.

^b^The values shown represent the mean of two independent biological replicates.
