# Supplementary material for: RNA-Seq Analysis Provides Insights for Understanding Photoautotrophic Polyhydroxyalkanoate Production in Recombinant Synechocystis Sp
Source: PLoS One. 2014 Jan 22;9(1):e86368. doi: 10.1371/journal.pone.0086368 (PMC3899235; doi:10.1371/journal.pone.0086368)
Supplement: Table S3 — Genes down-regulated in recombinant Synechocystis sp. strain CCsNphT7BCn (compared with CCsACnBCn)a. (DOCX) [file pone.0086368.s004.docx]

**Table S3 Genes down-regulated in recombinant *Synechocystis* sp. strain C_Cs_NphT7B_Cn_ (compared with C_Cs_A_Cn_B_Cn_)^a^**

| Gene ID | Description | Fold change | Expression level^b^ | | Functional category |
| --- | --- | --- | --- | --- | --- |
|  |  | (C_Cs_NphT7B_Cn_ vs C_Cs_A_Cn_B_Cn_) | C_Cs_A_Cn_B_Cn_ | C_Cs_NphT7B_Cn_ |  |
| slr0352 | transposase | -8.47 | 60.6 | 7.15 | DNA-binding |
| sll1560 | transposase | -6.26 | 107.19 | 17.12 | DNA-binding |
| slr2116 | spore coat polysaccharide biosynthesis protein ,SpsA | -4.96 | 89.07 | 17.97 | cell envelope |
| sll0709 | restriction enzyme LlaI protein | -4.82 | 200.58 | 41.63 | DNA repair |
| slr2031 | sigma factor SibG regulation protein, RsbU | -4.82 | 103.07 | 21.37 | metabolic process |
| slr2036 | transposase | -4.69 | 70.27 | 14.97 | DNA-binding |
| ssl2922 | virulence associated protein B | -3.85 | 22.7 | 5.89 | cellular process |
| sll0667 | transposase | -3.76 | 38.16 | 10.16 | DNA-binding |
| sll1998 | transposase | -3.71 | 68.76 | 18.54 | DNA-binding |
| slr2108 | polysialic acid transport ATP-binding protein, KpsT | -3.25 | 35.49 | 10.91 | lipopolysaccharide transport |
| sll1255 | transposase | -3.21 | 172.05 | 53.52 | DNA-binding |
| sll1408 | regulatory protein ,PcrR | -3.13 | 71 | 22.69 | transcription regulation |
| slr0704 | transposase | -3.05 | 11.72 | 3.84 | DNA-binding |
| slr0180 | transposase | -2.96 | 114.1 | 38.51 | DNA-binding |
| slr0323 | alpha-mannosidase | -2.94 | 103.54 | 35.25 | mannose metabolic process |
| slr1524 | transposase | -2.89 | 81.14 | 28.08 | DNA-binding |
| sll0092 | transposase | -2.86 | 268.41 | 93.91 | DNA-binding |
| sll0671 | magnesium and cobalt transport protein | -2.85 | 23.87 | 8.36 | cation transport |
| slr1803 | adenine-specific methyltransferase | -2.84 | 179.78 | 63.25 | DNA repair |
| sll1436 | transposase | -2.72 | 26.59 | 9.77 | DNA-binding |
| slr1860 | ICFG protein | -2.7 | 70.37 | 26.07 | metabolic process |
| sll1875 | heme oxygenase | -2.67 | 62.16 | 23.25 | porphyrin and chlorophyll metabolism |
| sll1983 | transposase | -2.66 | 26.91 | 10.13 | DNA-binding |
| slr1936 | transposase | -2.64 | 23.18 | 8.79 | DNA-binding |
| sll1685 | proton extrusion protein, PcxA | -2.62 | 15.29 | 5.85 | transport |
| slr1715 | transposase | -2.6 | 25.29 | 9.72 | DNA-binding |
| slr1523 | transposase | -2.51 | 119.94 | 47.7 | DNA-binding |
| sll1256 | transposase | -2.5 | 26.51 | 10.6 | DNA-binding |
| ssr2227 | transposase | -2.47 | 55.67 | 22.51 | DNA-binding |
| ssl0172 | transposase | -2.44 | 54.95 | 22.51 | DNA-binding |
| sll1871 | sensory transduction histidine kinase | -2.43 | 98.65 | 40.64 | signal transduction |
| slr1684 | transposase | -2.38 | 83.02 | 34.89 | DNA-binding |
| slr1585 | transposase | -2.32 | 33.31 | 14.36 | DNA-binding |
| sll1099 | elongation factor Tu | -2.3 | 660.41 | 287.17 | translation |
| slr1237 | cytosine deaminase | -2.27 | 196.04 | 86.18 | pyrimidine metabolism |
| sll1745 | 50S ribosomal protein L10 | -2.24 | 438.42 | 196.1 | translation |
| sll0729 | modification methylase | -2.24 | 41 | 18.34 | DNA repair |
| sll1596 | circadian rhythm protein | -2.22 | 87.08 | 39.21 | rhythmic process |
| sll1985 | transposase | -2.22 | 115.36 | 51.87 | DNA-binding |
| Sll0808 | transposase | -2.19 | 79.37 | 36.32 | DNA-binding |

^a^Only the top 40 highest decrease in fold-change and genes encoding known proteins are shown.

^b^The values shown represent the mean of two independent biological replicates.
