# Supplementary material for: RNA-Seq Analysis Provides Insights for Understanding Photoautotrophic Polyhydroxyalkanoate Production in Recombinant Synechocystis Sp
Source: PLoS One. 2014 Jan 22;9(1):e86368. doi: 10.1371/journal.pone.0086368 (PMC3899235; doi:10.1371/journal.pone.0086368)
Supplement: Table S4 — Strains and plasmids used in this study. (DOCX) [file pone.0086368.s005.docx]

Table S4 Strains and plasmids used in this study

| Strains or plasmids | Relevant characteristics | Source or reference |
| --- | --- | --- |
| Cyanobacteria strains | | |
| PCC 6803 | Wild-type strain of *Synechocystis* sp. PCC 6803, glucose tolerant | [1] |
| pTKP2031V | P_psbAII_::MCS integrated at *slr2031* in PCC 6803 genome | This study |
| C_Cs_A_Cn_B_Cn_ | P_psbAII_::*phaC_C_*_s_, *phaA_Cn_*, *phaB_Cn_* integrated at *slr2031* in PCC 6803 genome | This study |
| C_Cs_NphT7B_Cn_ | P_psbAII_::*phaC_C_*_s_, *nphT7*, *phaB_Cn_* integrated at *slr2031* in PCC 6803 genome | This study |
| *E*. *coli* strain |  |  |
| DH5α | F^-^, Φ80d*lacZ*Derutaemu15, delta (*lacZYA-argF*) U169, *DeoR*, *recA*1, *endA*1, *hsdR*17 (r_K_^-^, M_K_^+^), *phoA*, *supE*44, lambda^-^, *thi*-1, *gyrA*96, *relA*1 | Takara |
| Plasmid |  |  |
| pTKP2031V | Km^r^; f1 ori; P_psbAII_::MCS | [2] |
| pTKP2031V-*phaAB_Cn_* | Km^r^; f1 ori; P_psbAII_::*phaA_Cn_*, *phaB_Cn_* | This study |
| pTKP2031V-*phaC_C_*_s_*A_Cn_B_Cn_* | Km^r^; f1 ori; P_psbAII_::*phaC_C_*_s_, *phaA_Cn_*, *phaB_Cn_* | This study |
| pTKP2031V-*phaC_C_*_s_*nphT7phaB_Cn_* | Km^r^; f1 ori; P_psbAII_::*phaC_C_*_s_, *nphT7*, *phaB_Cn_* | This study |

Km^r^, kanamycin resistance

*phaC_C_*_s_ (*Chromobacterium* sp.), PHA synthase; *phaA_Cn_* (*C*. *necator*), β-ketothiolase; *phaB_Cn_* (*C*. *necator*), acetoacetyl-CoA-reductase; *nphT7* (*Streptomyces* sp.), acetoacetyl-CoA synthase

1. Williams JGK (1988) Construction of specific mutations in photosystem II photosynthetic reaction center by genetic engineering methods in *Synechocystis* 6803. Methods Enzymol 167:766-778.
2. Satoh S et al. (2001) Chlorophyll b expressed in cyanobacteria functions as light-harvesting antenna in photosystem I through flexibility of the proteins. J Biol Chem 276:4293-4297.
