# Supplementary material for: RNA-Seq Analysis Provides Insights for Understanding Photoautotrophic Polyhydroxyalkanoate Production in Recombinant Synechocystis Sp
Source: PLoS One. 2014 Jan 22;9(1):e86368. doi: 10.1371/journal.pone.0086368 (PMC3899235; doi:10.1371/journal.pone.0086368)
Supplement: Table S5 — Primers used in this study. (DOCX) [file pone.0086368.s006.docx]

Table S5 Primers used in this study

| Primers | Sequence ^a^ | Target gene |
| --- | --- | --- |
| Plasmids construction | | |
| *phaAB_Cn_* (F; *Nde*I) | GCCATATGCCAGCAATGGATGCAG | *C*. *necator* *phaA* and *phaB* genes |
| *phaAB_Cn_* (R; *Hpa*I) | TGGTTAACCGATTGCTACTTCCAT | *C*. *necator* *phaA* and *phaB* genes |
| *phaC_C_*_s_ (F; *Sfu*I) | GCTTCGAACCAGCAATGGATGCAG | *Chromobacterium* sp. *phaC* gene |
| *phaC_C_*_s_ (R; *Aat*I) | TGAGGCCTCGATTGCTACTTCCAT | *Chromobacterium* sp. *phaC* gene |
| *nphT7* (F; *Sfu*I) | TCTTCGAACTATTCACGACCATTTG | *Streptomyces* sp. *nphT7* gene |
| *nphT7* (R; *Aat*I) | CCGAGGCCTCGGACATCTGAGGTA | *Streptomyces* sp. *nphT7* gene |
| Real-time PCR | | |
| *phaC_S_*_s_ (F) | AAGGGGAAGTGATGATTGGCG | *Synechocystis* sp. *phaC* gene |
| *phaC_S_*_s_ (R) | CGGGGAAAGATTGGACGGTGT | *Synechocystis* sp. *phaC* gene |
| *phaC_C_*_s_ (F) | ACCGAAAAAGTCCACGAAAAGC | *Chromobacterium* sp. *phaC* gene |
| *phaC_C_*_s_ (R) | CGATGTAGGTCTCCCAAGTGAAG | *Chromobacterium* sp. *phaC* gene |
| *phaA_S_*_s_ (F) | CACCAGTCGGAAAGGAACCC | *Synechocystis* sp. *phaA* gene |
| *phaA_S_*_s_ (R) | GCGGTAATAGGCAACTCAGGAAA | *Synechocystis* sp. *phaA* gene |
| *phaB_S_*_s_ (F) | TCGAAGGCATGTATGAACGGA | *Synechocystis* sp. *phaB* gene |
| *phaB_S_*_s_ (R) | GTGTCAATGAAACCAGGGGCT | *Synechocystis* sp. *phaB* gene |
| *phaB_Cn_* (F) | AGGTCGGCGAGGTTGATGT | *C*. *necator* *phaB* gene |
| *phaB_Cn_* (R) | GCCTTGGCGGTGGAGTAGT | *C*. *necator* *phaB* gene |
| 16s rRNA (F) | GGACGGGTGAGTAACGCGTA | *Synechocystis* sp. 16S rRNA gene |
| 16s rRNA (R) | CCCATTGCGGAAAATTCCCC | *Synechocystis* sp. 16S rRNA gene |

^a^Restriction enzymes digestion sites are underlined.
